# Vascular and Neurological Complications in Sphenoid Sinusitis

**DOI:** 10.7759/cureus.66181

**Published:** 2024-08-05

**Authors:** Sanjay M Khaladkar, Sravya Julakanti, Ankita Pandey, Sayali Paidlewar, Ojasvi Sharma

**Affiliations:** 1 Radiodiagnosis, Dr. D. Y. Patil Medical College, Hospital and Research Centre, Dr. D. Y. Patil Vidyapeeth (Deemed to be University), Pune, IND

**Keywords:** infarct, dural venous sinus thrombosis, mastoiditis, subdural empyema, internal carotid artery stenosis, cavernous sinus thrombophlebitis, sphenoid sinusitis

## Abstract

Although the cavernous sinus and internal carotid artery are in close proximity to the sphenoid sinus, vascular complications in sphenoid sinusitis are rare due to the intervening mucosa and bone. Variations like dehiscence or aggressive infection can cause vascular complications, leading to cavernous sinus thrombosis, while perivascular inflammation of the internal carotid artery can result in stenosis or occlusion. Untreated or aggressive sphenoid sinusitis can cause neurological complications such as cerebral infarcts, meningitis, subdural empyema, cerebral abscess, and cranial nerve injuries. Magnetic resonance imaging (MRI) of the brain with angiography can depict these complications at an early stage. Additionally, mastoiditis can cause dural venous sinus thrombosis, which, if left untreated, can result in venous infarcts. We report a case of an 11-year-old male with sphenoid sinusitis who developed a left middle cerebral artery (MCA) territory infarct, cavernous sinus thrombophlebitis, subdural empyema, and meningitis. He also developed left transverse and sigmoid sinus thrombosis due to left mastoiditis.

## Introduction

Sphenoid sinusitis causing septic cavernous sinus thrombosis and internal carotid artery (ICA) stenosis or occlusion is a rare complication but can be catastrophic and cause irreversible sequelae. Mortality and morbidity rates associated with sphenoid sinusitis and its complications were higher in the pre-antibiotic era. The rate of residual neurological deficit is more than 30% [1,2].

Intracranial and intravascular complications in sphenoid sinusitis occur in less than 1% of cases. These include thrombophlebitis, orbital venous thrombosis, venous congestion, carotid vasculitis and narrowing or occlusion, meningitis, subdural empyemas, cerebral abscess (subdural, extradural, and cerebral), infarcts and cranial nerve injuries, and osteomyelitis. Subdural empyema is the most common complication (22-45%, mean 33%), followed by brain abscess (19-35%, mean 27%) and meningitis (15-26%, mean 20%). Complete recovery occurs in 61-81% of cases (mean 71%), while fatalities occur in 3-9% of cases (mean 6%) [3]. Early diagnosis of cavernous sinus thrombosis and ICA stenosis is difficult. Prompt administration of appropriate antibiotics and surgical drainage are important to reduce complications.

Our case had vascular complications due to concurrent sphenoid sinusitis and mastoiditis, including left cavernous sinus thrombosis and narrowing of the cavernous portion of the left ICA due to sphenoid sinusitis, as well as left transverse and sigmoid sinus thrombosis due to left mastoiditis. The patient also had a left middle cerebral artery (MCA) territory infarct, leptomeningitis, and cerebritis.

## Case presentation

An 11-year-old male child presented with complaints of a throbbing headache that had lasted for a duration of 15 days, which was associated with intermittent fever, nausea, and reduced appetite. There was also a history of neck pain for the same duration, along with three episodes of vomiting. His birth history was normal. Immunization and developmental milestones had been achieved as per age.

**Figure 1:**
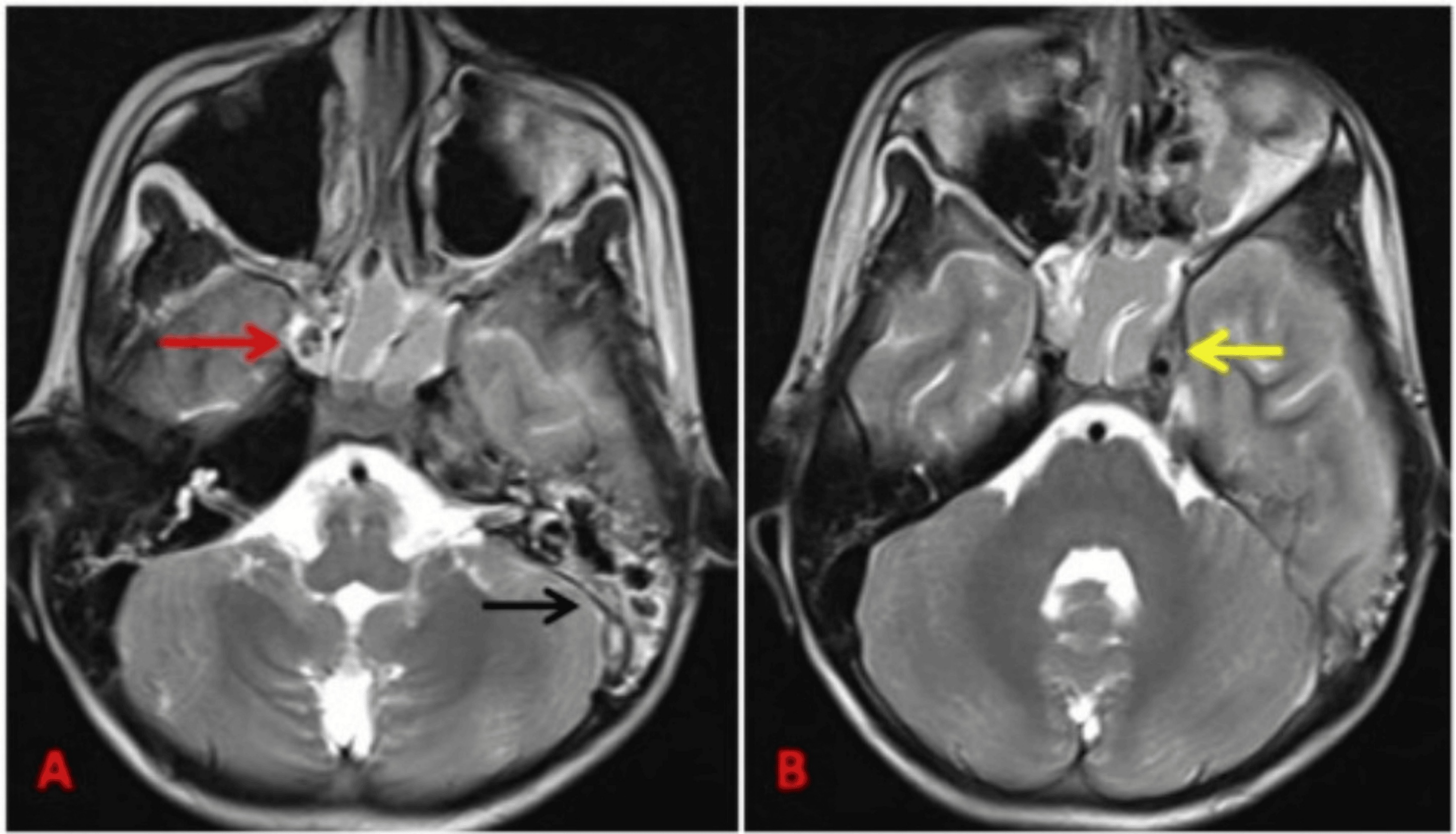
(A) Axial T2 showing sphenoid sinusitis appearing hyperintense on T2-weighted images with inspissated secretions appearing hypointense (marked by red arrow). Left mastoiditis showing hyperintense fluid collection on T2-weighted images (marked by black arrow). (B) Axial T2 showing bulky left cavernous sinus with outer convex margin (marked by yellow arrow).

**Figure 2:**
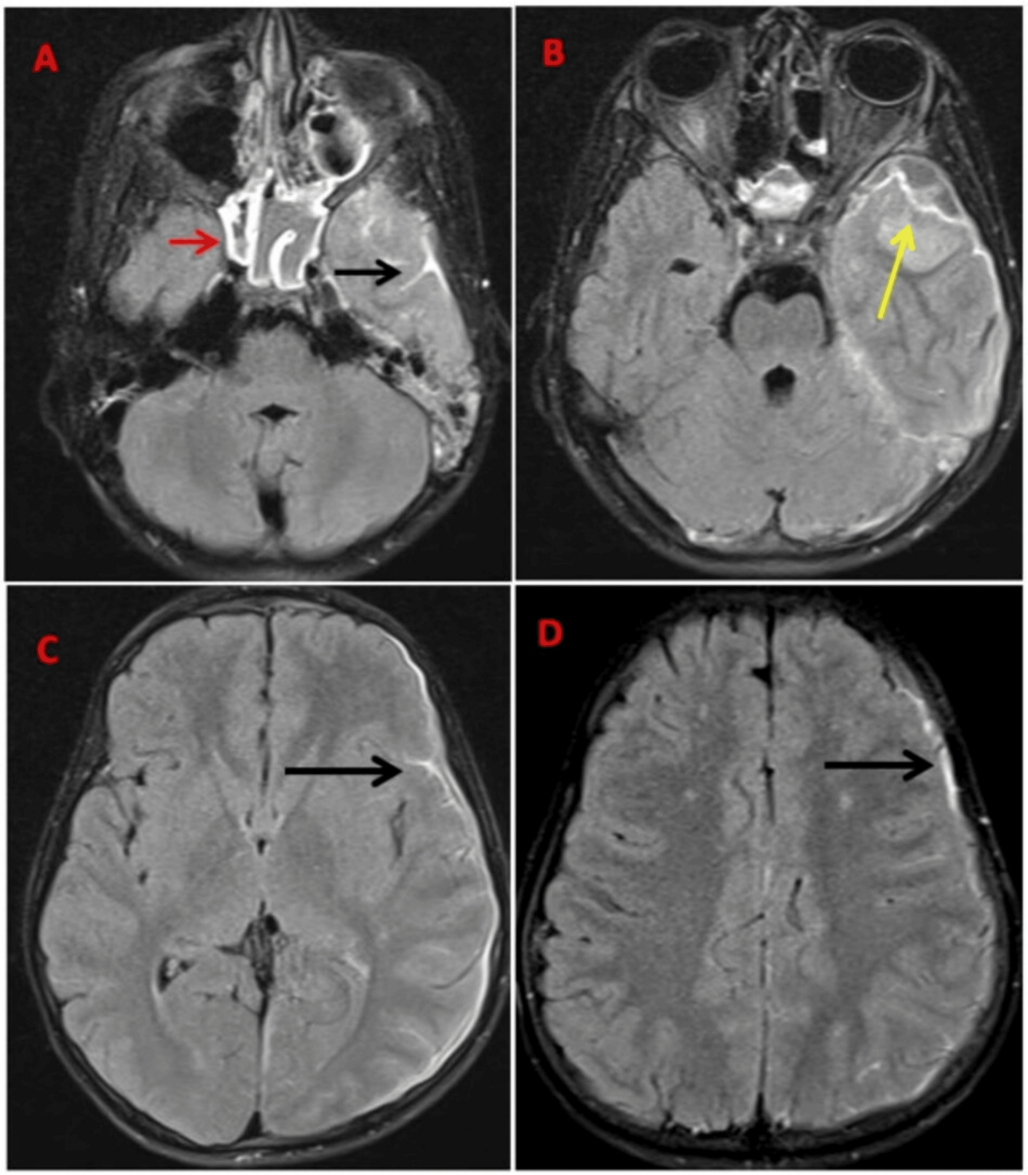
(A-D) Axial contrast FLAIR showing sphenoid sinusitis with mucosal enhancement (marked by red arrow), subdural empyema in the left fronto-temporo-parietal region (marked by yellow arrow), and pachymeningeal and leptomeningeal enhancement in the left temporo-parietal region (marked by black arrow). FLAIR: fluid-attenuated inversion recovery.

**Figure 3:**
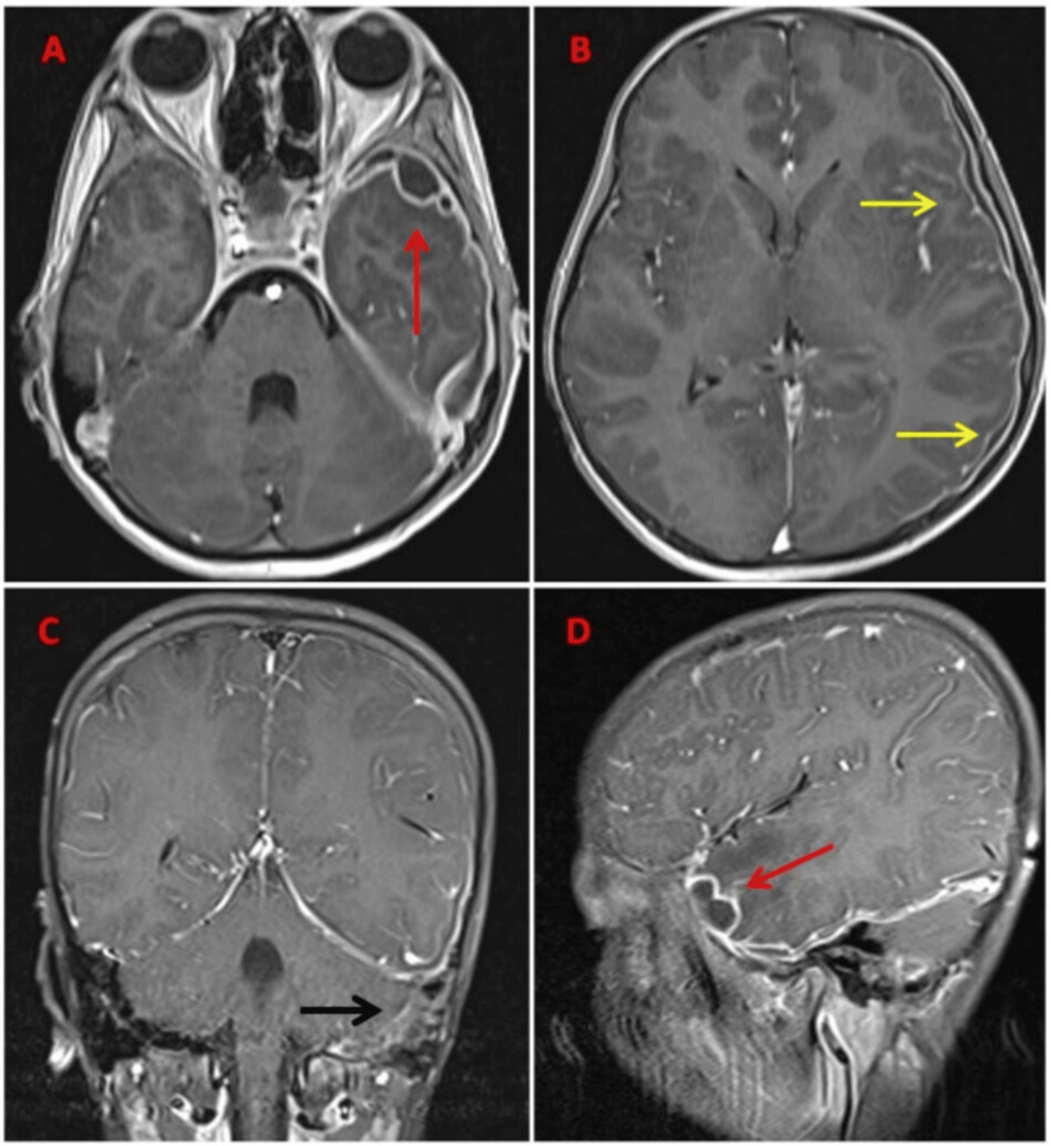
Post-contrast T1FS: (A-B) Axial, (C) Coronal, (D) Sagittal showing subdural empyema in the left temporal region (marked by red arrow), leptomeningeal enhancement in the left fronto-temporal region (marked by yellow arrow), and filling defect in the left sigmoid sinus (marked by black arrow). T1FS: T1 fat-saturated.

Magnetic resonance imaging (MRI) of the brain, both plain and contrast, was performed. The sphenoid sinus showed opacification appearing hyperintense on T2-weighted imaging (T2WI) with hypointense foci suggestive of inspissated secretions (Figures 1A, 2A). T2 hyperintense fluid collection was noted in the left middle ear cavity and mastoid air cells, suggestive of mastoiditis (Figure 1A). Heterogeneous enhancement was noted in the left cavernous sinus, which appeared bulky with an outer convex wall and showed diffusion restriction on diffusion-weighted imaging (DWI), suggestive of cavernous sinus thrombosis (Figure 1B). A loculated subdural fluid collection measuring approximately 27 x 10 x 19 mm (anteroposterior x transverse x craniocaudal) was noted in the left middle cranial fossa anterior to the temporal lobe appearing T1 hypointense, T2 hyperintense, and showing diffusion restriction on DWI with enhancement of the inner and outer margin in contrast study, suggestive of empyema (Figure 2B, 3A, 3D). A thin subdural collection (of 3 mm thickness) showing dense pachymeningeal and leptomeningeal enhancement was noted in the left fronto-temporo-occipital region (Figure 2C, 2D, 3B). The left leaf of the tentorium cerebelli showed thickening and enhancement. Loss of flow void was noted in the left transverse and sigmoid sinus extending to the proximal internal jugular vein (IJV) on T2WI, suggestive of venous sinus thrombosis (Figure 3C).

**Figure 4:**
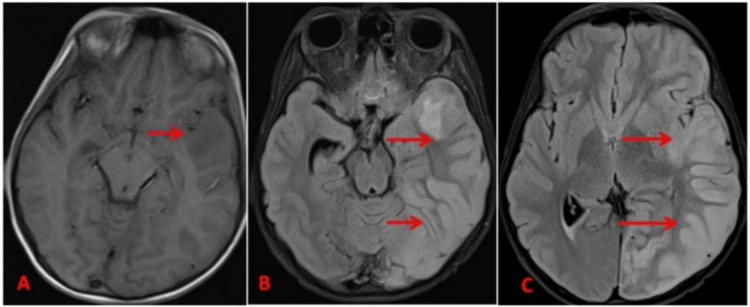
(A) Axial T1, (B-C) Axial FLAIR showing ill-defined hypointense signal in the left temporal lobe on T1-weighted images, appearing hyperintense on FLAIR (marked by red arrows). FLAIR: fluid-attenuated inversion recovery.

Enhancing exudates were noted in the sulcal spaces of the left fronto-temporo-parietal region with left hemispheric edema. An ill-defined T2 hyperintense lesion was noted in the left temporal lobe, predominantly involving white matter, appearing hypointense on T1-weighted images (T1WI) without foci of blooming on gradient echo imaging (GRE), not showing diffusion restriction on DWI, and without post-contrast enhancement, indicating either cerebritis or a venous non-hemorrhagic infarct (Figure 4A-C).

**Figure 5:**
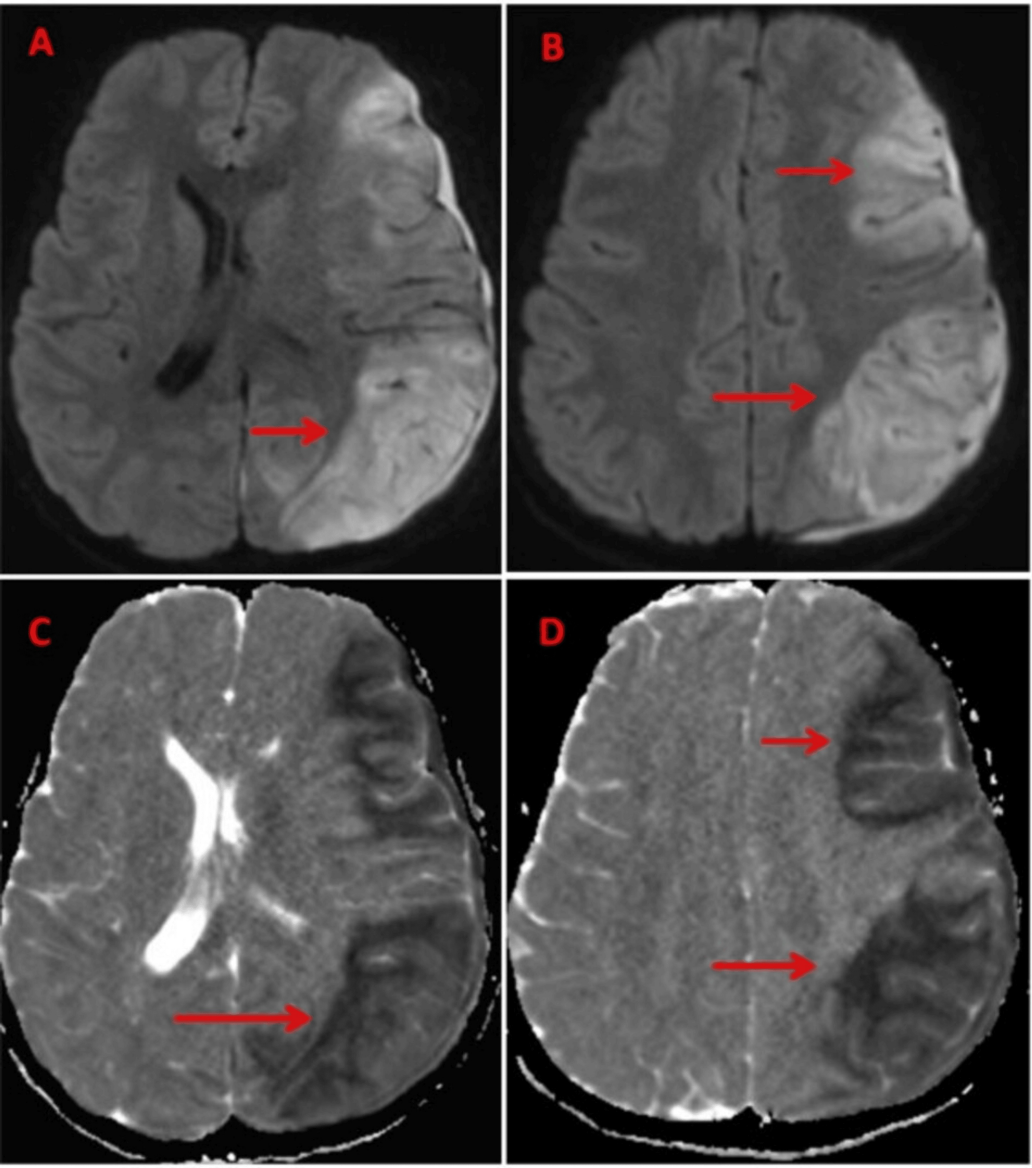
DWI (A-B) and ADC (C-D) showing a non-hemorrhagic infarct in the left fronto-parietal region, appearing bright on DWI with low ADC values (marked by red arrow). DWI: diffusion-weighted imaging, ADC: apparent diffusion coefficient.

A follow-up MRI of the brain was done two days later for signs of raised intracranial tension (early papilledema), right-sided deviation of the mouth, and irritability. A large ill-defined hyperintense lesion was noted on T2/fluid-attenuated inversion recovery (FLAIR) sequences involving both grey and white matter in the left fronto-temporo-parieto-occipital region, showing diffusion restriction without foci of blooming on GRE, suggestive of an acute non-hemorrhagic infarct (likely due to septic vasculitis or cerebritis) (Figure 5).

**Figure 6:**
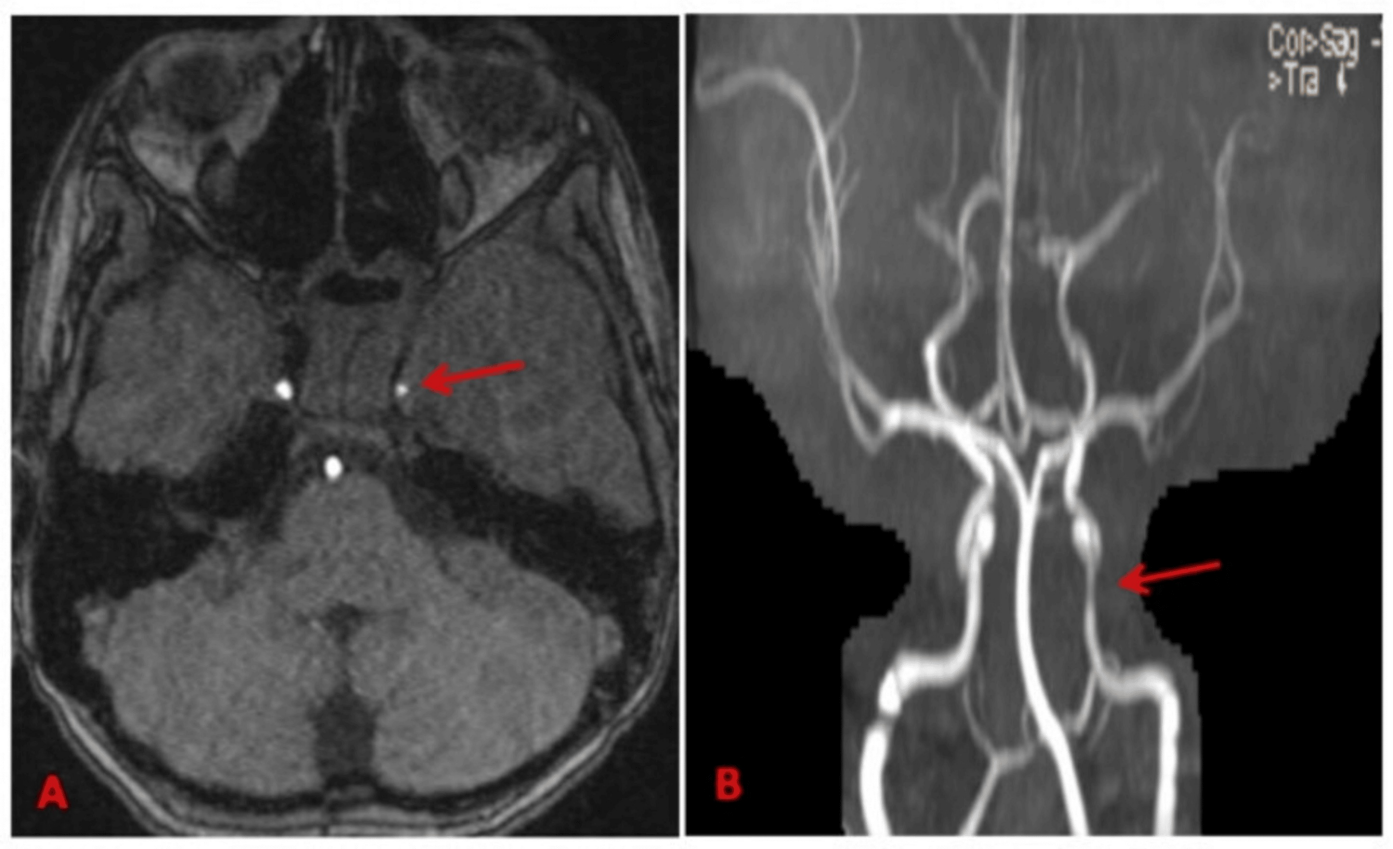
(A) 3D TOF, (B) MIP image showing narrowing of the cavernous portion of the left ICA (marked by red arrow). 3D TOF: three-dimensional time-of-flight, MIP: maximum intensity projection, ICA: internal carotid artery.

Mass effect was observed on the left lateral ventricle and third ventricle, causing a 6 mm shift of midline structures towards the right, along with early signs of uncal herniation. Other findings of the previous MRI were persistent. MRI brain angiography revealed narrowing of the cavernous portion of the left ICA and a paucity of vessels in the M3 and M4 segments of the left MCA (Figure 6).

**Figure 7:**
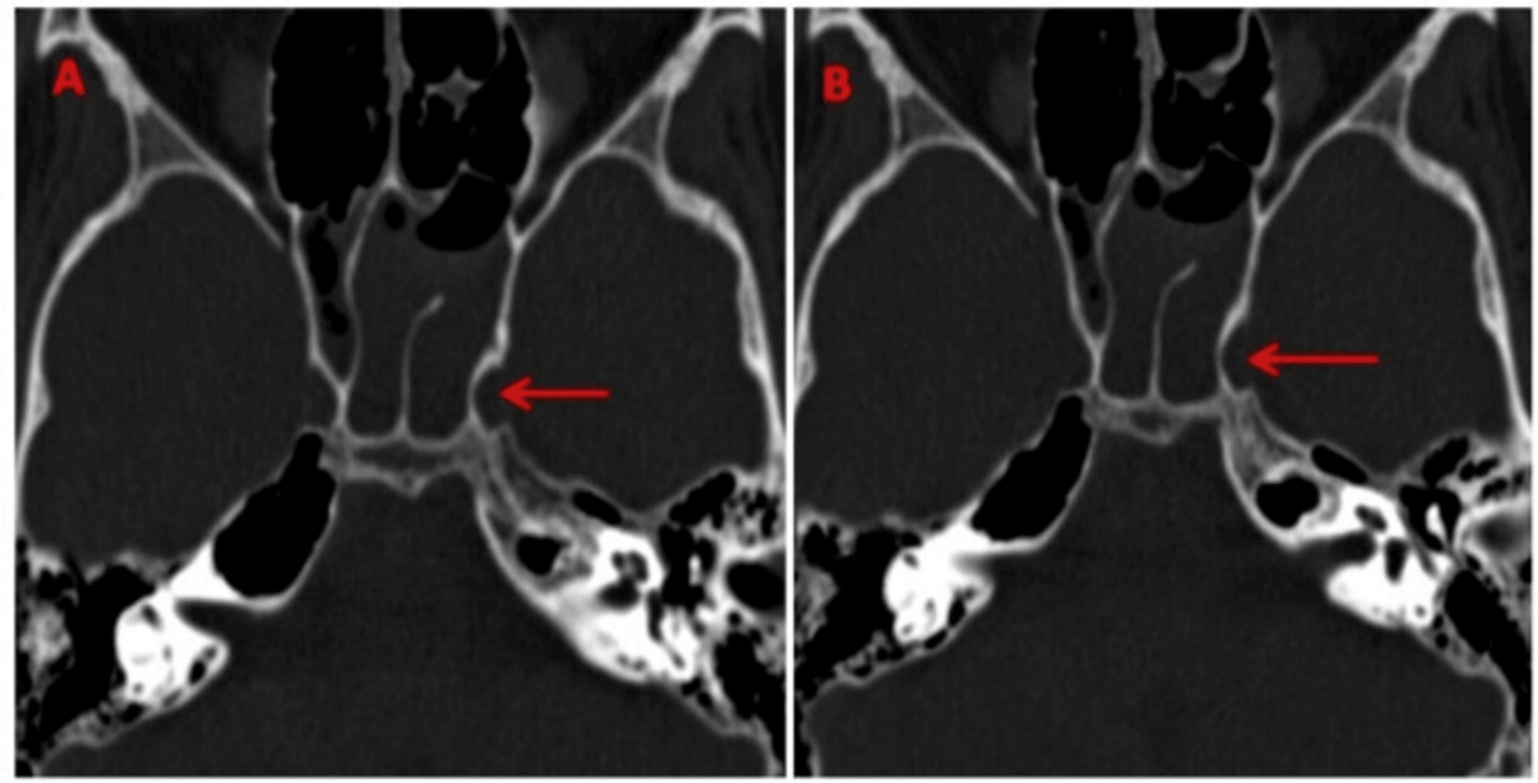
(A, B) HRCT base of skull showing proximity of the left carotid canal to the sphenoid sinus with protrusion of the carotid canal into the adjoining left sphenoid sinus (marked by red arrow). HRCT: high-resolution computed tomography.

These changes were likely due to unilateral protrusion of the ICA into the sphenoid sinus with direct involvement of the arterial wall due to sphenoid sinusitis causing arteritis (Figure 7).

The laboratory investigations revealed several significant abnormalities (Table 1). The high-sensitivity C-reactive protein (CRP-HS) level was highly elevated at 260 mg/L (normal range: less than 3 mg/L), and the erythrocyte sedimentation rate (ESR) was elevated at 26 mm/hr (normal range: 0-10 mm/hr, typically lower in children). These two parameters indicated a significant inflammatory response in the body. Homocysteine levels were normal at 5.34 µmol/L (normal range: 5.08-15.39 µmol/L). The blood tests revealed a significant increase in neutrophils at 87% (normal: 40-60%) and a decrease in lymphocytes at 6% (normal: 20-40%), indicative of polymorphonuclear leukocytosis. This, along with mild thrombocytosis and a total white blood cell count of 27,500/µL (normal range: 4,000 to 9,500/µL), strongly suggested a bacterial infection or other inflammatory process. The elevated platelet count of 533,000/µL (normal range: 150,000 to 410,000/µL) further supported the presence of an inflammatory or reactive condition. The D-dimer level was elevated at 1298 ng/mL (normal range: less than 500 ng/mL), which could indicate abnormal blood clotting activity. The partial thromboplastin time (PTT) was shortened at 18.60 seconds (normal range: 8.97 to 12.9 seconds), and the International Normalized Ratio (INR) was elevated at 1.53 (normal range: 0.85 to 1.15), both suggesting possible coagulation abnormalities. Antithrombin was below normal at 85 (normal range: 96-126), and Protein C was also below normal at 49 (normal range: 66-118), indicating potential issues with the body's anticoagulant mechanisms. Protein S was within the normal range at 105 (normal range: 65-140). Cerebrospinal fluid (CSF) analysis showed 10% neutrophils (normal: 0-6%), 80% lymphocytes (normal: 40-80%), and 10% macrophages (normal: 15-45%), with a glucose level of 31 mg/dL (normal: 40-80 mg/dL) and protein level of 113.2 mg/dL (normal: 15-45 mg/dL). The CSF appeared clear with no cobweb formation. This indicated infection.

**Table 1:** Blood Tests and Cerebrospinal Fluid Analysis Results CRP-HS: high-sensitivity C-reactive protein, ESR: erythrocyte sedimentation rate, PTT: partial thromboplastin time, INR: international normalized ratio, CSF: cerebrospinal fluid.

Parameter	Observed value	Normal value
CRP-HS	260 mg/L	<3 mg/L
ESR	26	0-10 mm/h (typically lower in children)
Homocysteine levels	5.34 µmol/L	5.08-15.39 µmol/L
Blood neutrophils	87%	40-60%
Blood lymphocytes	6%	20-40%
Total white blood cell count	27,500/µL	4,000 to 9,500/µL
Platelet count	533,000/µL	150,000 to 410,000/µL
D-dimer	1298 ng/mL	<500 ng/mL
PTT	18.60 seconds	8.97-12.9 seconds
INR	1.53	0.85-1.15
Anti-thrombin	85	96-126
Protein C	49	66-118
Protein S	105	65-140
CSF neutrophils	10%	0-6%
CSF lymphocytes	80%	40-80%
CSF macrophages	10%	15-45%
CSF glucose	31 mg/dL	40-80 mg/dL
CSF protein	113.2 mg/dL	15-45 mg/dL
CSF appearance	Clear, no cobweb formation	Clear, no cobweb formation

Tests for dengue IgG, IgM, and NS1 were negative. Liver function tests (LFT) and renal function tests (RFT) were normal, as were urine examination results. Tests for human immunodeficiency virus (HIV), hepatitis B surface antigen (HBsAg), and hepatitis C were non-reactive, and severe acute respiratory syndrome coronavirus 2 (SARS-CoV-2) IgG antibodies were negative. Hepatitis C virus (HCV) antibodies were also negative. Blood and urine cultures showed no growth of microorganisms, and CSF culture showed no acid-fast bacilli or microbial growth. The tuberculin test was negative. Fundoscopy performed two days after admission revealed bilateral early papilledema.

The diagnosis of sphenoid sinusitis with arteritis affecting the intra-cavernous portion of the ICA, left cavernous sinus thrombosis, cerebritis/non-hemorrhagic infarct affecting the left temporal lobe, a large infarct in the left fronto-temporo-parietal occipital region, meningitis, left mastoiditis, left transverse and sigmoid sinus thrombosis, and subdural empyema in the left fronto-temporo-parietal region, with left facial palsy, was made.

The patient received an intravenous broad-spectrum antibiotics regimen, including vancomycin 20 mg/kg/dose every six hours, meropenem 40 mg/kg/dose every eight hours, amikacin 15 mg/kg/dose once daily, ceftriaxone 15 mg/kg/dose every 12 hours, and doxycycline 2.2 mg/kg/dose every 12 hours. Antiepileptics such as phenytoin 5 mg/kg/day every 12 hours and piracetam 30 mg/kg/day every 12 hours were administered intravenously to prevent and control seizures, which can exacerbate neurological damage and worsen patient outcomes. He received intravenous mannitol 500 mg/kg every six hours for raised intracranial tension, along with other supportive measures. Additionally, intravenous steroids like dexamethasone 0.15 mg/kg/dose every six hours were administered to reduce inflammation and enhance antibiotic absorption. Anticoagulants like enoxaparin 1 mg/kg/dose every 12 hours were administered subcutaneously.

**Figure 8:**
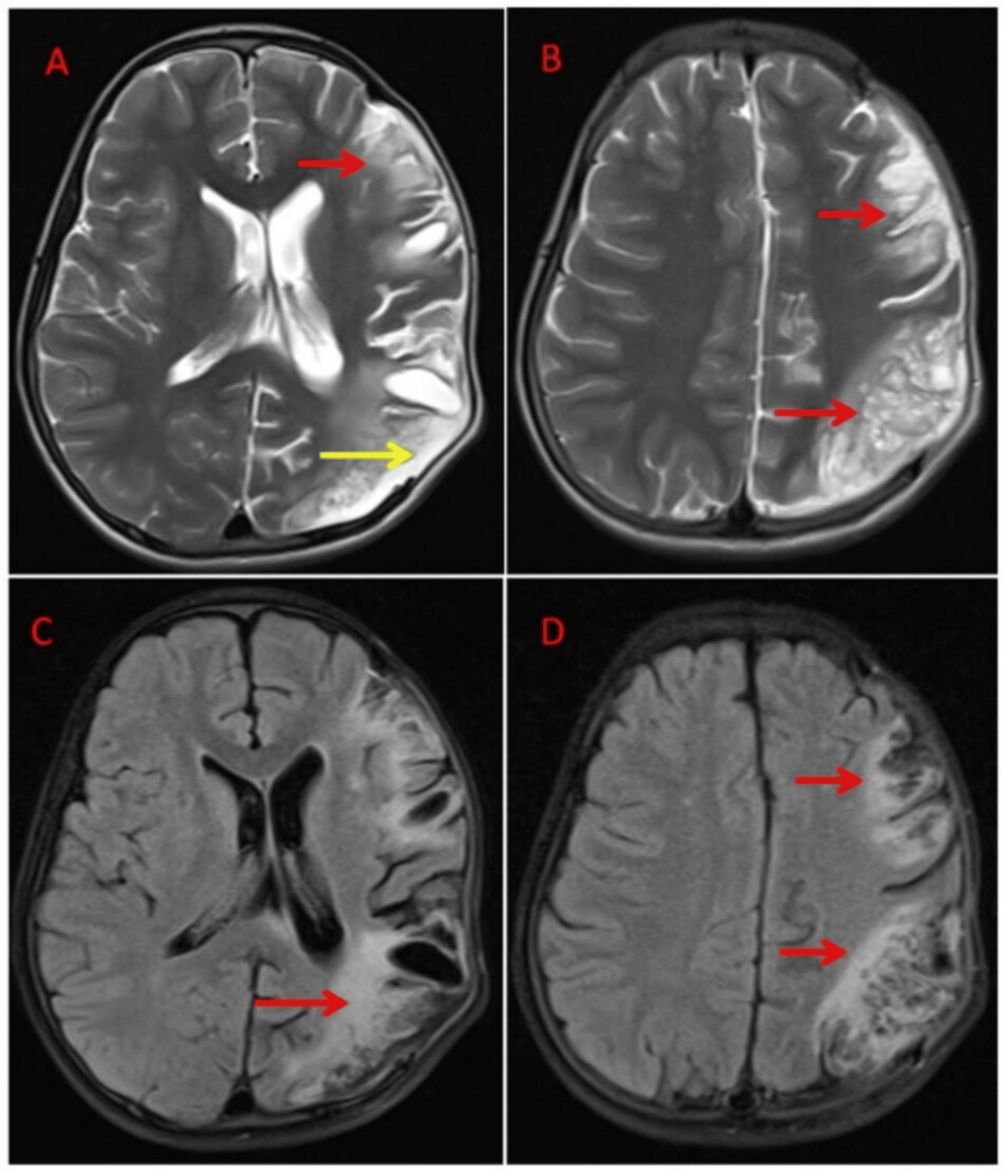
(A-B) Axial T2, (C-D) Axial FLAIR showing extensive areas of encephalomalacia following CSF intensity on T2/FLAIR sequences, with surrounding gliosis appearing hyperintense on T2/FLAIR in the left fronto-parietal region (marked by red arrows). A large craniectomy defect in the left fronto-parietal region with mild herniation of parenchyma (marked by yellow arrow). FLAIR: fluid-attenuated inversion recovery, CSF: cerebrospinal fluid.

Two days after the radiological examination, a left fronto-temporo-parietal decompression craniectomy was performed, along with excision of the subdural empyema. The purpose of this procedure was to diminish the bacterial presence and inhibit the advancement of vascular occlusion. Following the surgery, the patient showed improvement. A follow-up MRI of the brain three months later showed cystic encephalomalacia with gliosis in the region of the previous infarct (Figure 8).

Six months later, cranioplasty was performed. The patient was discharged on antibiotics and prophylactic antiepileptics and was asked to follow up 10 days later for suture removal. Histopathological examination of the excised dura mater showed a chronic inflammatory lesion, with no acid-fast bacilli or fungal hyphae detected.

## Discussion

The cavernous sinus is located adjacent to the walls of the sphenoid sinus and houses critical structures such as the ICA and the third, fifth, and sixth cranial nerves. Normally, it is separated from the sphenoid sinus by mucosa and bone. If sphenoid sinusitis becomes aggressive or untreated, it can extend into the cavernous sinus, leading to severe and irreversible neurological complications. These may include thrombophlebitis, venous congestion, orbital venous thrombosis, vasculitis affecting the carotid artery with narrowing or blockage, meningitis, subdural empyemas, cerebral abscesses, infarctions, and injuries to cranial nerves. Inflammation around the ICA can result from exposure to inflammatory substances present in retained infected sinus fluids and mucosa. The restricted diffusion observed on DWI in cerebral infections is attributed to the high viscosity, hypercellularity, and binding of water to macromolecules. Magnetic resonance angiography is a useful, non-invasive imaging modality for vascular stenosis [4].

As the ICA passes posterolateral to the sphenoid sinus wall, the absence or dehiscence of the bony wall separating the ICA from the sphenoid sinus makes it vulnerable to arterial involvement in sphenoid sinusitis and also vulnerable to high-risk injury during endoscopic surgery [5].

The average distance between the cavernous portions of the ICA is 12 mm. In 71% of cases, the ICA bulges into the sphenoid sinus. In 4% of cases, the ICA is exposed in the sphenoid sinus without bony protection. The distance between the carotid arteries ranges from 4 to 18 mm, with a mean of 12 mm [6]. Rarely, the cavernous ICA is as close as 4 mm to its counterpart, covered only by mucosa [7].

Wong et al. reported four patients with sphenoid sinusitis having ICA narrowing on MRI brain and magnetic resonance angiography. All four cases had narrowing of the cavernous segment of the ICA; three cases had narrowing in the supraclinoid segment of the ICA; one patient had a loss of flow-related signal in the bilateral proximal anterior cerebral artery (ACA) and MCA, and cerebral infarcts in two patients. The cavernous sinus in all cases showed heterogeneous enhancement; one case showed the presence of air in the cavernous sinus and one case showed diffusion restriction on DWI [4].

Sadineni et al. reported a case of a 32-year-old female who had presented with a fever and headache for 15 days, followed by left hemiparesis, blurred vision, diplopia, and left ophthalmoplegia for the past five days. Computed tomography (CT) followed by MRI brain with magnetic resonance angiography and contrast study showed findings of sphenoid sinusitis, osteomyelitis affecting the mid base of the skull, including the sphenoid bone, clivus, and occipital condyles. Additionally, there was an acute infarct in the right MCA territory due to infective vasculitis, stenosis of the petro-cavernous segment of the right ICA attributed to vasculitis, a mycotic aneurysm emerging from the cavernous portion of the left ICA, and thrombosis in the left cavernous and transverse sinuses, along with early signs of meningitis [8].

Chen et al. reported two cases of acute sphenoid sinusitis complicated by septic cavernous sinus thrombosis and ICA stenosis. In Case 1, a female patient had less extensive sinusitis with right-sided cavernous sinus thrombosis and critical ICA occlusion, along with acute ischemic changes noted at the basal ganglia on DWI. Case 2 involved a male patient with extensive pansinusitis, meningitis, cerebritis, and vasculitis attributed to a fungal infection, with lesser ICA stenosis. Both patients received broad-spectrum antibiotics and underwent surgical debridement. Additionally, Case 2 received antifungal medication [9].

In our case, sphenoid sinusitis led to left cavernous sinus thrombosis, narrowing of the cavernous portion of the left ICA, left MCA territory infarct, and meningitis. Associated left mastoiditis was observed. Left transverse and sigmoid sinus thrombosis was likely secondary to mastoiditis. A differential diagnosis of an acute non-hemorrhagic infarct can be cerebritis due to aggressive sphenoid sinusitis causing meningitis and a venous non-hemorrhagic infarct due to transverse sinus thrombosis.

Treatment typically begins with empiric broad-spectrum antibiotics initially, followed by adjustments based on culture results. It is essential to also rule out fungal causes. Surgical drainage is advised to reduce the bacterial load and mitigate the risk of advancing vascular occlusion. Corticosteroids help in reducing inflammatory damage and facilitate the passage of antibiotics across the blood-brain barrier. The use of anticoagulants is controversial. When facing ICA stenosis or occlusion, endovascular interventions such as balloon angioplasty and stent placement may not be suitable due to the vulnerability of the infected artery or its complete blockage. Literature reports supporting the use of external carotid-ICA bypass for septic cavernous sinus thrombosis-related occlusion or ICA stenosis are lacking. However, it may be a feasible option in stable or chronic stages for establishing adequate vascularization [9].

## Conclusions

Vascular complications in sphenoid sinusitis are rare but can cause severe neuro-deficits. A high index of suspicion is needed to diagnose cavernous sinus thrombosis and carotid artery involvement. MRI of the brain with angiography is extremely useful in diagnosing vascular complications and resultant neurological issues. This case illustrates the sequelae of vascular complications in both sphenoid sinusitis and mastoiditis.
